# Recommended centrifuge method: Specific grain size separation in the <63 µm fraction of marine sediments

**DOI:** 10.1016/j.mex.2024.102718

**Published:** 2024-04-20

**Authors:** E.J. Pryor, D. Tangunan, H.J.L. van der Lubbe, M.H. Simon, I.R. Hall

**Affiliations:** a School of Earth and Environmental Sciences, Cardiff University, United Kingdom; b Department of Earth Sciences, University College London, Gower Street, London WC1E 6BT, UK; c Department of Earth Sciences, Cluster Geochemistry & Geology, Vrije Universiteit Amsterdam (VU); d NORCE Norwegian Research Centre, Bjerknes Centre for Climate Research, Bergen, Norway

**Keywords:** Clay fraction, Lithogenic sediment, Biogenic carbonate rich sediment, Centrifugation, Grain size isolation, *Sediment grain size isolation of <63 µm fraction using a centrifuge*

## Abstract

The isolation of specific grain size classes of lithogenic samples and biogenic carbonate from the <63 µm fraction (i.e. clay and silt) of marine sediment is often a prerequisite to further pre-treatments and/or analytical measurements for palaeoceanographic studies. Established techniques employed have included sieving, settling and micro-filtration (and/or a combination of these). However, these methods often use significant amounts of bulk sediment (often up to ∼3 g) and/or require considerable amounts of time during sediment processing (ranging from 48 h to 3 weeks) to isolate a size specific class for further analyses. Here, we build on previous approaches to isolate three grain size classes (e.g. <2 µm, clay; 2–10 µm, fine silt; and 10–63 µm, coarse silt) from the <63 µm fraction of marine sediment with the aid of a centrifuge at varying revolutions per minute using Stokes’ Law. We show the utility of our approach using two common sediment types dominated by (i) lithogenic and (ii) biogenic carbonate (specifically coccoliths) components of marine sediment cores. Our method reduces the amount of sample material required to 1–2 g to provide an isolated clay fraction (or other targeted size fraction) and decreases the sample processing time (to ∼1 hour) to enable high throughput of analysis, when compared to previous techniques for palaeoceanographic proxy measurements.

• We recommend a more straightforward grain size isolation method for lithogenic sediment and biogenic carbonate sediment types
• Isolating commonly targeted grain size fractions for palaeoceanographic studies using a centrifuge

## Graphical abstract

**Figure:**
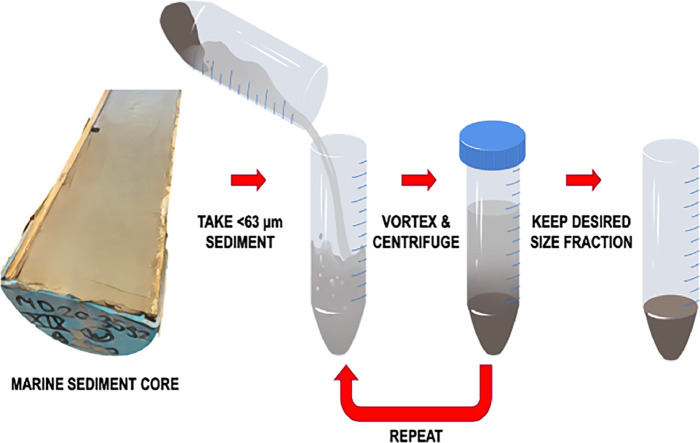

Specifications table

**Table:** 

Subject area:	Earth and Planetary Sciences
More specific subject area:	*Palaeoclimate and Geochemistry*
Name of your method:	*Sediment grain size isolation of <63 µm fraction using a centrifuge*
Name and reference of original method:	*N.A*
Resource availability:	*N.A*

## Introduction

Marine sediment cores provide long-lasting continuous archives of both marine and terrestrial environmental conditions. These past environmental conditions can be inferred via palaeoproxy measurements that record geochemical and biological information, for example, via key geochemical elemental ratios indicative of a specific environmental variable (e.g. [1], [2], [3], [4]), or the relative abundances of species that have specific ecosystem tolerances (e.g. [5,6]), such as a change in climate conditions. A requirement of many palaeoproxy measurements is the ability to quantitatively isolate specific grain size classes (e.g. <2 µm clay fraction) from a mixed <63 µm sediment sample without significant modification of their chemical or physical properties. For instance, marine sediment provenance studies (i.e. the identification of specific geographic source areas of sediment) require the exact separation of grain size classes prior to analyses, because different transport processes may contribute preferentially to specific grain size fractions [7,8]. For example, a key tool used to identify sediment provenance are radiogenic isotopes (mainly neodymium (εNd) and strontium (^87^Sr/^86^Sr)) of the lithogenic clay fraction [7,9]. There is an ongoing effort to determine the extent to which ^87^Sr/^86^Sr isotope ratios are sensitive to grain size variability and chemical weathering [7,[10], [11], [12], [13]]. It is widely recognised that the ^87^Sr/^86^Sr composition of marine sediment typically increases with decreasing grain size [11], since clay minerals are preferentially derived from Rb-rich minerals such as biotite and K-feldspars, which are more prone to chemical rather than physical weathering processes [14,15]. The grain size sensitivity of ^87^Sr/^86^Sr isotope compositions has caused inconsistencies when comparing different core sites used for proxy-based provenance reconstructions. Hence, limitations persist concerning the utility of Sr isotope data from the bulk sediment fraction, or when comparing existing data from different grain size fractions ([7,[16], [17], [18], [19]]; van der Lubbe, 2016; [20]). For example, Hahn et al. [16] identified a + 0.02 - offset in ^87^Sr/^86^Sr isotope data measured in the <2 µm fraction compared to <120 µm fraction, 0.00001 (10^−5^) orders of magnitude higher than the ^87^Sr/^86^Sr isotope variability usually considered significant [21]. Unlike ^87^Sr/^86^Sr isotope ratios, the εNd signatures are less sensitive to chemical weathering and thus grain size variation [7]. For palaeoclimate reconstructions, the influence of different grain size fractions on provenance analyses needs to be carefully accounted for, especially when interpreting land-sea linkages [7]. Thus, as a prerequisite for such studies, the accurate separation of the grain size classes is essential.

Accurate and precise particle size separation is also an essential requirement during sample preparation for studies focused on the microfossil content of marine sediments. For example, coccolithophores (2 to ∼30 µm) are among the marine organisms that provide proxies for palaeoceanographic reconstructions from both organic and inorganic fossil records [22]. Particular organic biomarkers, i.e. alkenones, which are produced by a specific small-sized group of coccolithophores (e.g. *Emiliania huxleyi* and *Gephyrocapsa oceanica*) are important proxy of past sea surface temperature and productivity [23,24], as well as of atmospheric carbon dioxide (pCO_2_) concentrations [25]. Likewise, the geochemistry of coccoliths – coccolithophores inorganic exoskeletons made of calcite – provide a unique signal for past variations in surface water oceanographic and productivity conditions [26,27]. For example, the coccolith fraction (<20 µm) Sr/Ca is a well-developed proxy, reflecting coccolithophore growth rates and therefore documents variations in coccolithophore production independent of any coccolith counting [28]. The isotopic signals obtained from coccoliths is dependent on the size and the species, through vital effects [29,30] and species-specific growth rate [28], therefore isolating bulk coccoliths into consistent size fractions is necessary.

A wide range of techniques have been used to separate different grain size fractions from a bulk sediment sample. These include: 1) wet and dry sieving (e.g. [31,32]), 2) micro-filtration (e.g. [33]), 3) settling and decanting (e.g. [34]). These methods typically require significant amounts of bulk sediment (often up to ∼3 g) [35] and/or require considerable amounts of time during sediment processing [35,34]. Additionally a two-step centrifugation (e.g. [[35], [36]]) has been applied using different centrifuge speeds, followed by decantation and settling. This method successfully isolates the <2 µm fraction but takes 48 h so is significantly longer than our proposed 1-hour method.

For the biogenic fraction, the utility of coccolith trace element and isotopic analyses is still limited by the challenges of separating coccoliths from bulk sediments, especially the removal of the finer size fraction (<2 µm). Each technique has its own advantages, micro-filtration and settling are highly effective in isolating the larger size coccoliths (e.g. >3 µm) while centrifugation has been suggested to be more efficient in separating the finest particle (<3 µm; [37]). The efficacy of microfiltration diminishes when dealing with reduced amounts of sediments as separating the coccoliths into different precise size fractions (e.g. >12 µm, 10–12 µm, 8–10 µm, 5–8 µm, 3–5 µm, >2 µm) using a vacuum pump has its own limitations. The large diameter of the filter and the pressure exerted by the vacuum pump from the bottom can lead to the rupture of the membrane filter, rendering the technique impractical for precise size separation. Additionally, separating all the size fractions using a centrifugation method alone yielded low accuracy, where mixing of different coccolith sizes has been recorded [37]. Removing the <2 µm prior to doing the size separation of larger fraction for coccolith clumped isotopes, for example, will minimise, if not completely remove, diagenetic imprint in the samples. The presence of abiotic or diagenetic calcite has been reported to result in temperature offset between UK37 and Δ47-derived temperature from the 2–5 µm fraction [38]. The <2 µm particles, which were described to be calcitic [38] and could be identified as micarb [39], if not completely removed, could account for discrepancies between UK37- and Δ47-based sea surface temperature estimates. Therefore, any improvement in the size separation technique used in size-dependent coccolith analyses should focus on improving the precision and accuracy of the size class separation and the time required for processing. We provide a standardised protocol with a low time cost and small sample size of the initial sediment for both biogenic and lithogenic sediment components.

### Example 1: - Pre-treatment of lithogenic fraction (decarbonated)

Marine sediment from marine core site MD20–3591 (36° 43.707 S; 22° 9.151 E, water depth 2464 m) [40] and International Ocean Discovery Program (IODP) Site U1476 [41] located at the northern entrance of the Mozambique Channel (15°49.25′S; 41°46.12′E) was initially wet sieved at <63 µm to separate the coarse and fine sediment fraction. Before the grain size separation, approximately 1–2 g of the <63 µm freeze-dried sediment fraction was decarbonated, and organics and Fe-Mn coatings were removed following a method developed by previous studies (e.g. [[8], [19], [34], [42], [43]]). The separation of particles smaller than 2 µm was carried out using 5 mL 0.5 % sodium metaphosphate and 15 mL of deionised (DI) water, then centrifuging following Stokes’ Law (see full method (Table 1)).

### Example 2: - Pre-treatment of biogenic carbonate fraction

We used sediment materials from the IODP Site U1475 [41] drilled from the southwestern flank of the Agulhas Plateau (41°25.61′S; 25°15.64′E) spanning the mid-Pliocene (2.8 to 3.3 Ma). The site was chosen because of the dominance of the different sizes of the coccolithophore genus *Reticulofenestra* (>2 to ∼12 µm), which altogether comprise up to ∼90 % of the total assemblage, making the site an ideal study location for the size separation technique. Other subtropical to tropical species such as *Coccolithus pelagicus* (∼4 to 15 µm), *Helicosphaera* spp. (∼7 to 11 µm), and *Pseudoemiliania lacunosa* (∼4 to 7 µm) commonly co-occur in the sample.

A total of 16 samples were processed. About 1 g of freeze-dried bulk sediment was lightly crushed with a pestle and mortar, suspended into a 20 mL 0.5 % ammonia solution, disaggregated, and was then mixed using a vortex. The solution was then placed in a 50 mL centrifuge to separate the particles smaller than 2 µm following Stokes’ Law, assuming a density of 2.65 g/cm^3^ (see full method (Table 1)).

We provide an updated method of separating grain sizes using a centrifuge which is straightforward to follow and is time and cost-efficient. Minimal material is needed (1–2 g of <63 µm dry sediment) which means there is no bulk sample wasted. To confirm the utility of the grain separation technique, we have used grain size analysis for the lithogenic sediment and a combination of automated and manual counting techniques for the biogenic carbonate components. This separation technique improves how effectively the size separation works for both lithogenic sediment and biogenic carbonate (specifically coccoliths) components as well as offering the potential of retrieving sufficient sample material for future analyses such as clumped isotope and radiocarbon measurements in coccoliths, as well as other biogenic components (e.g. diatoms). Given radiogenic isotopes datasets reflect analyses on a variety of size fractions, this standardised size separation method will also lead to more comparable radiogenic isotopes datasets with consistent size fraction separation.

## Method details

### Method validation

#### Experimental design

Here, we explain the experimental design and tests used to isolate precise grain size classes from the <63 µm fraction into separate size classes. Our main focus is on the use of centrifugation as it provides the shortest processing times and is the most suitable approach for high throughput sample processing necessary to support proxy reconstructions that require many samples (typically high-resolution or long timescale studies). We will provide examples of the application of this method using lithogenic and biogenic carbonate sediment in separate work flows (Table 1).

**Table 1:** Method for isolation of <2 µm fraction^a^.

Step	Lithogenic	Biogenic Carbonate
1	Add 5 mL sodium metaphosphate solution (0.2 %), 15 mL DI water to tube 1.	Add 20 mL 0.5 % ammonia solution.
2	Vortex at 3000 rpm until fully suspended then centrifuge 1000 rpm, 2 min for fractions ∼2 µm.	
3	Decant solution of tube 1 into tube 2 and place tube 2 aside.	
4	Pour 5 mL Calgon solution (0.2 %), 15 mL DI water into tube 1 again.	Pour 20 mL 0.5 % ammonia solution into tube 1 again.
5	Vortex tube 1 until fully suspended then centrifuge 1000 rpm, 2 min.	
6	Pour solution of tube 1 into tube 2. Tube 2 contains 40 mL now.	
7	Vortex tube 2 until fully suspended then centrifuge 1000 rpm, 4 min, a. This removes the >2 µm fraction (settled) and the suspended solution contains the <2 µm clay fraction.	
8	Pour supernatant into tube 3, this contains the <2 µm clay fraction.	
9	Vortex tube 3 until fully suspended then centrifuge for 10 min at 2500 rpm.	Discard Tube 3.
10	Rinse samples using DI water (40 mL) three times.	Take tube 2. This contains the >2 µm fraction to be processed for further size separation (2–3 µm, 3–5 µm, 5–8 µm, 8–10 µm, 10–12 µm) using the micro-filtration technique.
11	After cleaning, place samples in tubes in an oven at 40 °C. a. Or can pipette sample onto a glass slide under the fume hood and allow to air dry for XRD work.	

a To isolate the 2–10 µm and 10–63 µm fraction we utilised Eq. (1), assuming a typical grain density of quartz (2.65 g/cm^3^), to determine the following centrifugation steps: i) 1000 rpm for 2 min isolating the 10–63 µm fraction, ii) using the supernatant, 1000 rpm for 2 min, iii) pour solution into a new centrifuge tube and 1000 rpm for 4 min. The resulting supernatant contains the <2 µm and 2–10 µm. iv) 2500 rpm for 10 min separates the 2–10 µm from <2 µm (Fig. 4).

#### Experimental set-up

According to Stokes' Law, the settling velocity of a particle is proportional to the density difference between the particle type and the liquid phase, inversely proportional to the viscosity of the liquid, and proportional to the square of the particle diameter. Equally, within a centrifuge, Stokes’ Law can be used to calculate the time of rotation required to separate specific size fractions according to Eq. (1) [44] which describes the relationship between time (T) and different components of the centrifuge and material being processed.

(1) T=9ηln(R2/R1)/8π2N2r2(ρ−ρ0)+2(ta+td)/3

Where:

T total time (*sec*)

η viscosity (poises)

R2 final distance from rotation axis (cm)

R1 initial distance from rotation axis (cm)

N angular velocity (rev/*sec*) r particle radius (cm)

*ρ* density of particle (g/cm^3^)

*ρ*0 density of displaced media (g/cm^3^)*

ta = time of acceleration (*sec*)

td = time of deceleration (*sec*)

* *We use the grain density of quartz (2.65 g/cm^3^).*

To determine the specific centrifuge settings for our experiment, we first consider the effect of the different key variables in Eq. (1), these are, a) grain density parameter, *ρ* and b) angular velocity, N. We additionally test the initial sample size to determine the effect on the isolation of the clay fraction. All experiments were carried out using a Thermo Scientific™ Sorvall™ ST 16 Centrifuge Series. We selected the following initial settings: R1 = 6 cm, R2 = 17 cm, acceleration = 5.26 s and deceleration = 15.2 s, according to Eq. (1). These parameters were designed to reduce sediment processing time.

##### Grain density parameter (*ρ*)

The lithogenic composition of most marine sediment is dominated by light minerals, mainly quartz (grain density = 2.65 g/cm^3^) and feldspar (2.57 to 2.77 g/cm^3^). The dominance of these light minerals results in average grain densities for marine sediments of about 2.65 g/cm^3^ for most grains > 63 µm (i.e. sand). This grain density assures the removal of the majority of the quartz grains if a 2 µm separation is carried out, although some particles of less dense minerals could remain in suspension [44]. Considering the higher grain density for calcite particles (2.71 g/cm^3^) we calculate the difference in centrifuge time, relative to a quartz grain density, to be ∼0.3 s in our experiments. Given this increase in centrifuge time is negligible compared to the time of centrifuge deceleration time (∼15.2 s) we applied a grain size density of 2.65 g/cm^3^ for calculating the centrifuge times for all experiments.

##### The angular velocity parameter (N)

We tested changing parameter N, the angular velocity, for various spins when separating the finest fraction (2 µm) from the lithogenic sediment samples. Adjusting the angular velocity modified the total spin time (T), resulting in either a time too low for the centrifuge setting (<1 min), or increasing the processing time. The revolutions per minute (rpm) tested were 500, 1000 and 2000, and all tests resulted in a successful size fraction isolation for the lithogenic sediment samples. We therefore set the angular velocity to 1000 rpm for the first three steps of separation to maximise the low processing time.

##### Sediment material- initial sample size

We tested the initial sample size (weight) of the <63 µm dry lithogenic sediment fraction necessary to result in a final weight of 20–50 mg for the <2 µm fraction of lithogenic sediment- a requirement for radiogenic isotope analysis [9]. Through testing different starting weights of dry <63 µm sediment, we typically find that a starting weight of 1–2 g of dry <63 µm sediment results in a sufficient amount of <2 µm fraction after centrifugation for subsequent analysis such as radiogenic isotope analysis.

### Application/Verification of the centrifugation set-up

The values obtained in the *Experimental Design* using Eq. (1) have helped determine specific settings for the new proposed method. We use samples from two common marine sediment types dominated by (i) lithogenic (ii) biogenic carbonate (specifically coccoliths) to test our protocol. For both sediment types, we demonstrate the utility of our protocol by isolating three grain size classes, the <2 µm, 2–10 µm and 10–63 µm fractions from the total <63 µm material, with minor uncertainty using Eq. (1). We test these specific size fractions as they are commonly used in palaeoceanographic studies (e.g. [7]); however, we emphasise the specific grain size fractions can easily be varied using Eq. (1).

For both lithogenic and biogenic sediment types, we tested the accuracy of the updated centrifuge method calculations for larger size fractions (2–10 µm and 10–63 µm). To isolate the 2–10 µm and 10–63 µm fraction we utilised Eq. (1) and values obtained in the *Experimental Design* testing to determine the centrifugation steps, assuming a typical grain density of quartz (2.65 g/cm^3^).

#### Grain size analysis of lithogenic fraction (decarbonated) for method validation

**Fig. 1:**
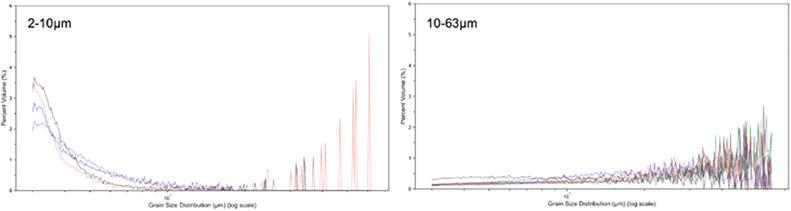
Grain size distributions from lithogenic sediment grain size separation; 2–10 µm (left), 10–63 µm (right). Colours indicate different sediment samples from the same core site (MD20–3591). For the 2–10 µm separated sediment, most of the sediment grain size distribution is within the 2–10 µm fraction with some outliers > 10 µm, these are single grains based on the data output (see data availability). *Note*: grain size distribution on log scale.

We measured the grain size distribution of the lithogenic sediment to confirm the size isolation was successful. For the 2–10 µm and 10–63 µm size fractions, we use Beckman Multisizer IV Coulter Counter and for the <2 µm size fractions we used the HELOS laser particle analyser because clays are platy and difficult to distinguish with the Beckman Multisizer IV Coulter Counter. Following the proposed standardised protocol, we find that over 90 % of the grains between 2 and 10 µm were isolated and over 70 % of the grains 10–63 µm were successfully isolated based on grain size analysis using the Beckman Multisizer IV Coulter Counter (Fig. 1). The percentage of isolation for the 2–10 µm and 10–63 µm fraction was calculated by summing the % volume of the particles within the respective size class.

**Fig. 2:**
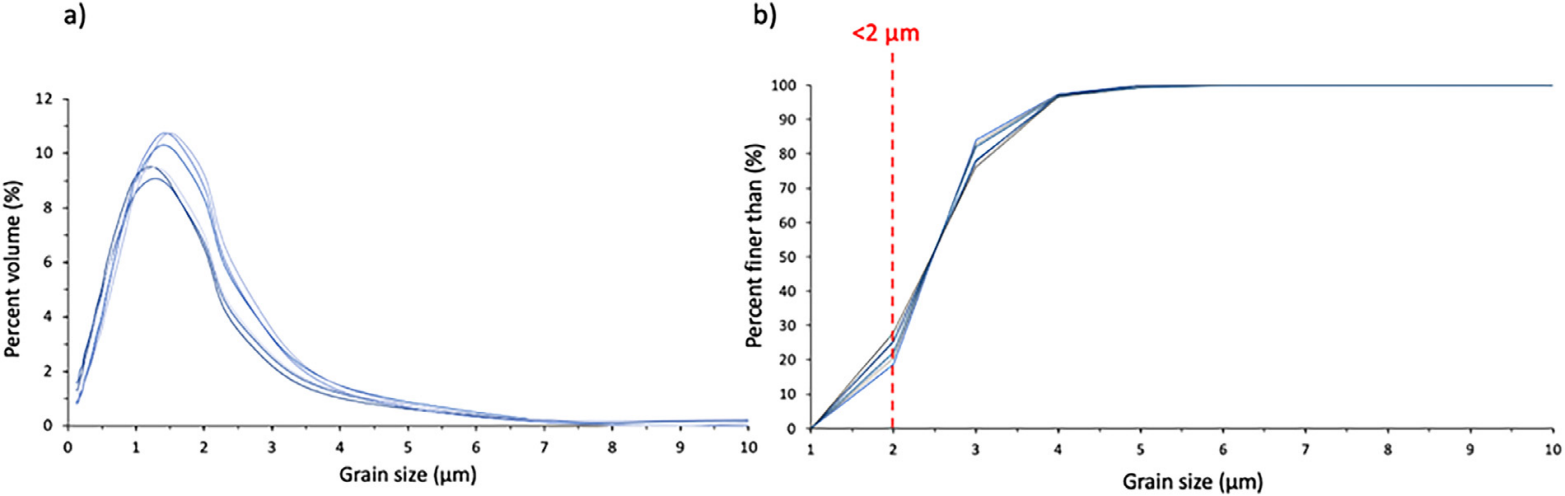
Separated clay fraction distributions. a) Grain size distribution of separated lithogenic clay fraction (<2 µm), b) Percent finer than with <2 µm threshold indicated (red dashed line).

Additionally, the <2 µm fraction was successfully isolated, with over 90 % of the sediment isolated recording as clay (<2 µm) using the HELOS laser particle analyser (Fig. 2a and b).

#### Biogenic carbonate fraction grain size separation validation

**Fig. 3:**
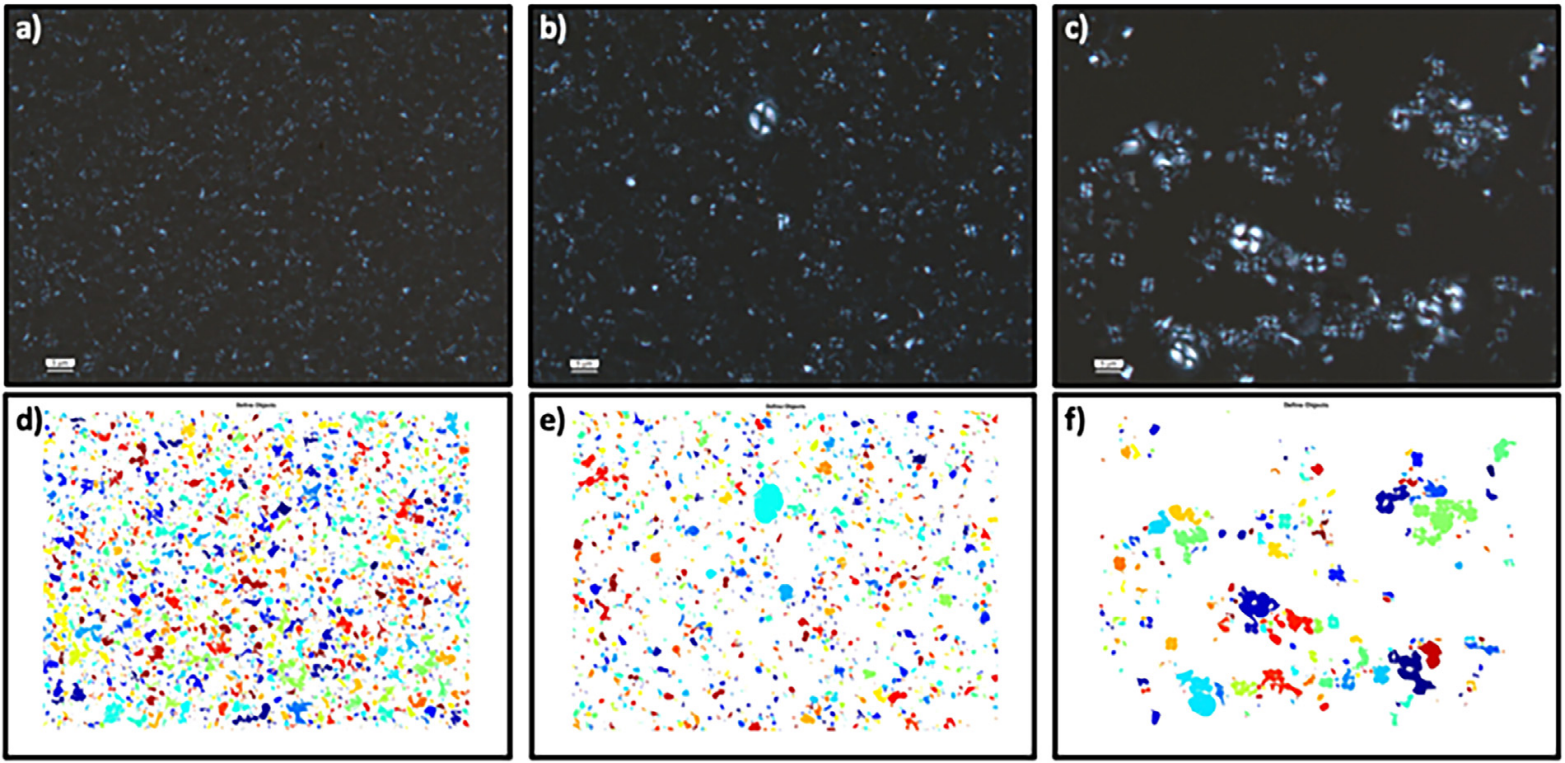
Images of biogenic sediment (top)- size fraction separation. a) <2 µm fraction, b) 2–10 µm fraction, c) 10–63 µm fraction. Tracing boundaries image analysis (bottom), each unjoined colour shape represents a different particle defined by the analysis. d) <2 µm fraction, e) 2–10 µm fraction, f) 10–63 µm fraction.

To determine whether the grain size isolation was successful in the biogenic carbonate fraction, we performed count analysis on the microscope images (Fig. 3), this is because grain size analysis using the Beckman Multisizer IV Coulter Counter or HELOS laser particle analyser requires the removal of carbonates and organics which implicates future coccolith analysis in biogenic sediment. Therefore, we prepared smear slides of the separated grain size fractions from biogenic carbonate sediment for microscopy (<2 µm, 2–10 µm and 10–63 µm). A total of 20 images for each size fraction were taken using the Leica DMLB transmitted light microscope fitted with Leica Application Suite imaging software. We carried out count analysis on MATLAB^Ⓡ^ 2019a using the image processing toolbox, by tracing the boundaries of grains within a microscope image (of a set size). We ensured that similar image size and resolution was used across the images as the minimum grain size depends on the pixel size. We created a binary image and removed background noise from the image. By having an original image and the binary image, it is possible to count the objects and perform image analysis, through subtracting the difference in the image contrast. The analysis reveals 48 % of the grains are 10–63 µm within the separated 10–63 µm class and for the samples separated to 2–10 µm, 68 % of the grains are 2–10 µm. For the <2 µm fraction, 45 % of grains were <2 µm and no coccoliths were present in the <2 µm isolated fraction (Fig. 3a and d).

## Recommended method

**Fig. 4:**
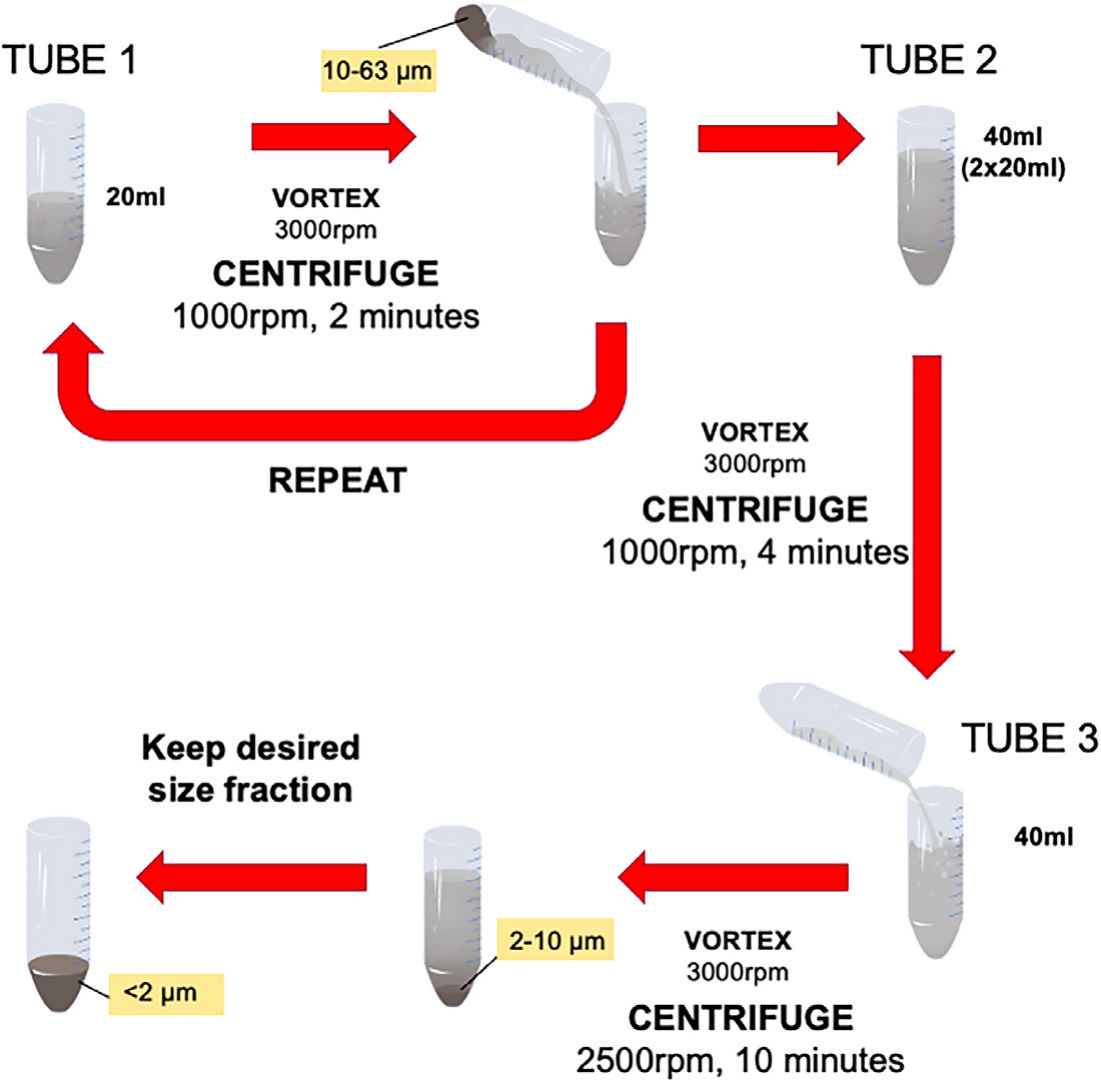
Our method for separating the <2 µm, 2–10 µm and 10–63 µm fractions through calculations using Eq. (1) for both lithogenic and biogenic carbonate sediment types.

Based on the experimental set up and centrifugation verification steps tested, we outline a recommended method for the separation of the <2 µm size fraction. For a detailed list of materials and equipment, please see Supplementary Information. The updated method for the separation of the <2 µm fraction discussed in this paper focuses on Step 6 (lithogenic) and Step 3 (biogenic carbonate) and is detailed in Fig. 4 and Table 1. *NB: tube refers to 50 mL centrifuge tube*.

Pre-treatment of marine sediment for lithogenic fraction:

1) Take 1–2 g of dry sediment <63 µm fraction (sieved),
2) Removal of carbonates,
3) Removal of organics,
4) Removal of eventual remaining carbonates,
5) Leach of Fe-Mn coatings of the lithogenic fraction,
6) **Centrifugation to isolate <2µm fraction using 50mL centrifuge tubes (see**Table 1**for in detail steps),**
7) Drying of samples,
8) Total sample digestion for radiogenic isotope analysis.

Pre-treatment of marine sediment for biogenic carbonate fraction:

1) Take 1–2 g of dry <63 µm fraction (sieved),
2) Add 40 mL 0.5 % ammonia solution,
3) **Centrifugation to isolate <2µm fraction using 50mL centrifuge tubes (see**Table 1**for in detail steps).**

## Conclusions

We provide an updated method of separating grain sizes using a centrifuge which is straightforward to follow and is time and cost-efficient. Previous techniques used for size separation include sieving, settling and micro-filtration, however our separation method relies on standard equipment such as a centrifuge because this approach provides the shortest processing times compared to previous techniques which can take 48 h - 3 weeks. Importantly, this method takes less than 1 hour to separate the varying size fractions and requires 1–2 g of starting <63 µm sediment. This amount of starting sediment is much less than previous methods which use a significant amount of starting sediment (up to ∼3 g). This separation technique improves how effectively the size separation works for both lithogenic sediment and biogenic carbonate (specifically coccoliths) components as well as offering the potential of retrieving sufficient sample material for future analyses such as clumped isotope and radiocarbon measurements in coccoliths, as well as other biogenic components (e.g. diatoms). Given radiogenic isotopes datasets reflect analyses on a variety of size fractions, this standardised size separation method will also lead to more comparable radiogenic isotopes datasets with consistent size fraction separation.

## Data availability

Grain size separation data from the HELOS laser particle analyser and the Beckman Multisizer IV Coulter Counter in this paper is available at DOI 10.5281/zenodo.10462213. The MATLAB^Ⓡ^ code for image processing was written by Trauth [45] (MATLAB^Ⓡ^ Recipes for Earth Sciences).

## Code availability

MATLAB^Ⓡ^ code for image processing from Trauth [45] (MATLAB^Ⓡ^ Recipes for Earth Sciences).

## CRediT authorship contribution statement

**E.J. Pryor:** Conceptualization, Methodology, Investigation, Data curation, Funding acquisition, Writing – review & editing, Writing – original draft. **D. Tangunan:** Conceptualization, Methodology, Investigation, Data curation, Supervision, Funding acquisition, Writing – review & editing, Writing – original draft. **H.J.L. van der Lubbe:** Conceptualization, Methodology, Supervision, Writing – review & editing. **M.H. Simon:** Conceptualization, Supervision, Writing – review & editing. **I.R. Hall:** Supervision, Writing – review & editing.

## Declaration of Competing Interest

The authors declare that they have no known competing financial interests or personal relationships that could have appeared to influence the work reported in this paper.

## Appendix Supplementary materials

Image, application 1

## References

[1] Caley T., Kim J.H., Malaizé B., Giraudeau J., Laepple T., Caillon N. (2011). High-latitude obliquity as a dominant forcing in the Agulhas current system. Climate of the Past.

[2] Diekmann B., Kuhn G., Rachold V., Abelmann A., Brathauer U., Fütterer D.K. (2000). Terrigenous sediment supply in the Scotia Sea (Southern Ocean): response to Late Quaternary ice dynamics in Patagonia and on the Antarctic Peninsula. Palaeogeogr. Palaeoclimatol. Palaeoecol..

[3] Møller H.S., Jensen K.G., Kuijpers A., Aagaard-Sørensen S., Seidenkrantz M.S., Prins M. (2006). Late-Holocene environment and climatic changes in Ameralik Fjord, southwest Greenland: evidence from the sedimentary record. Holocene.

[4] Stuut J.B.W., Kasten S., Lamy F., Hebbeln D. (2007). Sources and modes of terrigenous sediment input to the Chilean continental slope. Quat. Int..

[5] Lynch-Stieglitz J., Stocker T.F., Broecker W.S., Fairbanks R.G. (1995). The influence of air-sea exchange on the isotopic composition of oceanic carbon: observations and modeling. Global Biogeochem. Cycles.

[6] Spero H.J., Bijma J., Lea D.W., Bemis B.E. (1997). Effect of seawater carbonate concentration on foraminiferal carbon and oxygen isotopes. Nature.

[7] Meyer I., Davies G.R., Stuut J.B.W. (2011). Grain size control on Sr-Nd isotope provenance studies and impact on paleoclimate reconstructions: an example from deep-sea sediments offshore NW Africa. Geochem. Geophys. Geosyst..

[8] Stuut J.B.W., Prins M.A., Schneider R.R., Weltje G.J., Fred Jansen J.H, Postma G (2002). A 300-kyr record of aridity and wind strength in southwestern Africa: inferences from grain-size distributions of sediments on Walvis Ridge, SE Atlantic. Mar. Geol..

[9] van der Lubbe J.H.J.L., Frank M., Tjallingji R., Ralph R.S. (2016). Neodymium isotope constraints on provenance, dispersal, and climate-driven supply of Zambezi sediments along the Mozambique Margin during the past ∼45,000 years. Geochem. Geophys. Geosyst..

[10] Blum J.D., Erel Y. (2003). Radiogenic isotopes in weathering and hydrology. Treatise Geochem..

[11] E.J. Dasch, Strontium isotopes in weathering profiles, deep-sea sediments, and sedimentary rocks, 33 (1969), 1521–1552.

[12] Feng J.L., Zhu L.P., Zhen X.L., Hu Z.G. (2009). Grain size effect on Sr and Nd isotopic compositions in eolian dust: implications for tracing dust provenance and Nd model age. Geochem. J..

[13] Grousset F.E., Rognon P., Coudé-Gaussen G., Pédemay P. (1992). Origins of peri-Saharan dust deposits traced by their Nd and Sr isotopic composition. Palaeogeogr. Palaeoclimatol. Palaeoecol..

[14] Garzanti E., Padoan M., Setti M., López-Galindo A., Villa I.M. (2014). Provenance versus weathering control on the composition of tropical river mud (southern Africa). Chem. Geol..

[15] Jung S.J.A., Davies G.R., Ganssen G.M., Kroon D. (2004). Stepwise Holocene aridification in NE Africa deduced from dust-borne radiogenic isotope records. Earth Planet. Sci. Lett..

[16] Hahn A., Compton J.S., Meyer-Jacob C., Kirsten K.L., Lucasssen F., Pérez Mayo M. (2016). Holocene paleo-climatic record from the South African Namaqualand mudbelt: a source to sink approach. Quat. Int..

[17] Jewell A.M., Drake N., Crocker A.J., Bakker N.L., Kunkelova T., Bristow C.S. (2021). Three North African dust source areas and their geochemical fingerprint. Earth Planet. Sci. Lett..

[18] Kunkelova T., Crocker A.J., Jewell A.M., Breeze P.S., Drake N.A., Cooper M.J. (2022). Dust sources in Westernmost Asia have a different geochemical fingerprint to those in the Sahara. Quat. Sci. Rev..

[19] Simon M.H., Babin D.P., Goldstein S.L., Cai M.Y., Liu T., Han X. (2020). Development of a protocol to obtain the composition of terrigenous detritus in marine sediments -a pilot study from International Ocean Discovery Program Expedition 361. Chem. Geol..

[20] van der Lubbe J.H.J.L., Tjallingii R., Prins M.A., Brummer G.J.A., Jung S.J.A., Kroon D., Schneider R.R. (2014). Sedimentation patterns off the Zambezi River over the last 20,000 years. Mar. Geol..

[21] Biscaye P.E., Dasch E.J. (1971). The rubidium, strontium, strontium-isotope system in deep-sea sediments: argentine Basin. J. Geophys. Res..

[22] Stoll H.M., Ziveri P. (2004).

[23] Brassell S.C., Eglinton G., Marlowe I.T., Pflaumannt U., Sarntheint M. (1986). Molecular stratigraphy: a new tool for climatic assessment. Nature.

[24] Müller P.J., Kirst G., Ruhland G., Von Storch I., Rosell-Melé A (1998). Calibration of the alkenone paleotemperature index U37K based on core-tops from the eastern South Atlantic and the global ocean (60°N-60°S). Geochim. Cosmochim. Acta.

[25] Jasper J.P., Hayes J.M. (1990). A carbon isotope record of CO2 levels during the late quaternary. Nature.

[26] Beaufort L., Lancelot Y., Camberlin P., Cayre O., Vincent E., Bassinot F., Labeyrie L. (1997). Insolation cycles as a major control of equatorial Indian Ocean primary production. Science.

[27] Stoll H.M., Schrag D.P. (2000). High-resolution stable isotope records from the upper cretaceous rocks of Italy and Spain: glacial episodes in a greenshouse planet?. Bulletin of the Geological Society of America.

[28] Stoll H.M., Ziveri P. (2002). Separation of monospecific and restricted coccolith assemblages from sediments using differential settling velocity. Mar. Micropaleontol..

[29] Bolton C.T., Stoll H.M., Mendez-Vicente A. (2012). Vital effects in coccolith calcite: cenozoic climate-pCO2 drove the diversity of carbon acquisition strategies in coccolithophores?. Paleoceanography.

[30] Godbillot C., Minoletti F., Bassinot F., Hermoso M. (2022). Parallel between the isotopic composition of coccolith calcite and carbon levels across Termination II: developing a new paleo-CO2 probe. Clim. Past.

[31] Buller A.T., Mcmanus J. (1972). Modes of turbidite deposition deduced from grain-size analyses. Geol. Mag..

[32] Gee G.W., Bauder J.W. (1986). Particle-size Analysis. Methods Soil Anal. Part 1: Phys. Mineralogical Methods.

[33] Minoletti F., Hermoso M., Gressier V. (2009). Separation of sedimentary micron-sized particles for palaeoceanography and calcareous nannoplankton biogeochemistry. Nat. Protoc..

[34] Simon M.H., Babin D.P., Goldstein S.L., Cai M.Y., Liu T., Han X. (2020). Sequential extraction procedure to obtain the composition of terrigenous detritus in marine sediments. MethodsX.

[35] Bayon G., Toucanne S., Skonieczny C., André L., Bermell S., Cheron S. (2015). Rare earth elements and neodymium isotopes in world river sediments revisited. Geochim. Cosmochim. Acta.

[36] Stumpf R. (2011).

[37] Zhang H., Liu C., Mejía L.M., Stoll H. (2021). Technical note: accelerate coccolith size separation via repeated centrifugation. Biogeosciences.

[38] Drury A.J., John C.M. (2016). Exploring the potential of clumped isotope thermometry on coccolith-rich sediments as a sea surface temperature proxy. Geochem. Geophys. Geosyst..

[39] Rousselle G., Beltran C., Sicre M.A., Raffi I., De Rafelis M. (2013). Changes in sea-surface conditions in the Equatorial Pacific during the middle Miocene-Pliocene as inferred from coccolith geochemistry. Earth Planet. Sci. Lett..

[40] VAZQUEZ RIVEIROS Natalia, WAELBROECK Claire (2020) MD 225 /ACCLIMATE-2 cruise, RV Marion Dufresne. 10.17600/18001350.

[41] Hall I.R., Hemming S.R., LeVay L.J. (2016). International ocean discovery program expedition 361 Preliminary report: south African climates (Agulhas LGM density profile). Integr. Ocean Drilling Program: Preliminary Rep..

[42] Bayon G., German C.R., Boella R.M., Milton J.A., Taylor R.N., Nesbitt R.W. (2002). An improved method for extracting marine sediment fractions and its application to Sr and Nd isotopic analysis. Chem. Geol..

[43] Gutjahr M., Frank M., Stirling C.H., Klemm V., van de Flierdt T., Halliday A.N. (2007). Reliable extraction of a deepwater trace metal isotope signal from Fe-Mn oxyhydroxide coatings of marine sediments. Chem. Geol..

[44] Hathaway J.C. (1955). Procedure for clay mineral analyses. Clay Miner. Bull..

[45] Trauth M. (2021).

