# Supplementary material for: Recommended centrifuge method: Specific grain size separation in the <63 µm fraction of marine sediments
Source: MethodsX. 2024 Apr 20;12:102718. doi: 10.1016/j.mex.2024.102718 (PMC11041909; doi:10.1016/j.mex.2024.102718)
Supplement: Supplementary file 1 [file mmc1.docx]

*Supplementary Information*

The following includes the equipment and materials needed for the updated centrifuge method (see Table 1):

*Equipment:*

- Centrifuge
- Vortex
- Hot plate
- Polarising microscope

*Hardware/glassware:*

- Reagent bottles (1000 mL/2000 mL)
- Centrifuge tubes (50 mL)
- De-ionised (DI) water squirt bottle (250 mL)
- Microscope slide glass
- Microscope cover slip

*Chemicals used in this procedure:*

**For lithogenic:**

- 2M Acetic acid
- Buffered (with sodium acetate) acetic acid (1M)
- Hydrogen peroxide (6%)
- 0.5% sodium metaphosphate solution (i.e. Calgon)
- Hydroxylamine Hydrochloride leach
- DI water

**For biogenic carbonate:**

- DI water
- 0.5% ammonia solution
